# Supplementary material for: Kernel graph filtering—A new method for dynamic sinogram denoising
Source: PLoS One. 2021 Dec 2;16(12):e0260374. doi: 10.1371/journal.pone.0260374 (PMC8638912; doi:10.1371/journal.pone.0260374)
Supplement: Attachment — Submitted filename: Reviewer_comments.pdf [file pone.0260374.s002.pdf]

Review of the manuscript entitled "*Kernel graph filtering - a new method for dynamic sinogram denoising*" by Guo *et al.*, submitted for publication to PLOS ONE.

General comment:

The denoising of PET data has always been the center of attention in the field of medical imaging. There are many studies in the current literature regarding this issue. In this paper, the authors introduced the kernel graph filtering method for denoising PET sinograms. The idea behind this work is interesting. However, the explanation of the theory employed in this method is confusing to me. Some of the parameters are not defined properly and I cannot go through the derivations carefully. The authors need to re-write these once again with clear explanation. I attached my detailed comments. Considering my comments, I recommend major revision of the manuscript before being considered for publication in PLOS ONE.

Major comments:

1) The methods part is confusing. Not enough physical explanation has been provided for the employed theoretical model.

2) Lines 70-71, is index "i" is essentially the scan time? Please explain.

3) Line 81, what does the sigma of matrix W represent? Please explain. It is confusing to me.

4) Line 86, what is the physical meaning of the parameter "$\boldsymbol{x}$". Please define and explain.

5) Lines 73-75, "The sinogram denoising method to be proposed in this paper does not require specific knowledge about the corrupting noise in the noisy sinograms. Therefore there is no further assumption on pi's.", meaning is unclear. Please re-write.

6) Equation (1), the Gaussian kernel function, the 2 parameter Gaussian function could be represented as;

$$G_{2D}(x,y) = \frac{1}{2\pi\sigma^2} e^{\left(-\frac{x^2+y^2}{2\sigma^2}\right)}$$

Comment on the similarities and differences of your Gaussian kernel function and the one I showed above.

7) What does α represent in Eq. (3)? I am again confused.

8) Equation (5), what γ actually does here?

9) Lines 141-144, "At smaller i's, the energy is low and energy variations are large, we use smaller knni to include fewer neighbour sinograms, namely, fewer nonzero aij's. At larger i's, the energy is high and energy variations are small, we use larger knni to include more neighbour sinograms, namely, more nonzero aij's". I am again confused here, and I cannot follow the derivations. Please re-write.

10) Please discuss in detail and in a clear manner, the difference between your work and previous work cited in Ref.[10] by Wang *et. al*.

11) The PET data you have used are actually simulated and you have introduced Poisson noise, the method seems to work well. However, it is highly recommended to use realistic experimental data. Please use some realistic measurement examples and compare.

12) Lines 267-275, the authors used some parameter values. I am again confused how these values are chosen? Some sort of justification would be needed for the use of these values. If in case, it is really not possible to justify and these values are chosen in a "random" fashion, the you need to perform sensitivity study by changing the values of each of these parameters and investigate their respective influence on the final results.

I have some PET data of mouse head, there are namely two file extension .img and .hdr, does your program model takes .img and .hdr? please confirm this.

Minor comments:

1) Lines 14-15, "For example, when other imaging modalities, such as magnetic resonance (MR) or computed tomography (CT), are incorporated, some bone or MR-only lesion [4] originally nonexistent in the PET image may be introduced.". meaning unclear, please re-write.

2) Line 24, "…correlated and the is very difficult to reduce." Remove "the".

3) Line 31, "…to suppress, and spur the development…", please consider replacing the word "spur" with something else.

4) Lines 31-32, "It has been shown that pre-denoising in sinograms more efficient post-denoising in PET images". Wrong grammar, re-write.

5) Regarding MLEM method, please consider citing more recent papers. Example;

Studies on unfolding energy spectra of neutrons using maximum-likelihood expectation-maximization method. Nuclear Science and Techniques. 2019 Sep;30(9):1-0.

6) Lines 227-228, "To get dynamic PET images, the time activity curve (TAC) in Fig. 1 (b) was filled into corresponding tissues to produce the noiseless ground truth images". Please explain how you have obtained the time-activity curves in your work.

7) Lines 269-270, "For fair comparison, these parameters were tuned differently on the sinograms data". How did you tune these parameters? Please elaborate.

8) Fig. 6, Please consider changing line and symbol colors for different method, it is hard to differentiate these. Check for similar issue in other plots.

9) In conclusion, Lines 447-448, “Extensive simulation studies and tests on in-vivo dynamic PET data have shown the efficacy and advantages of the proposed method over the existing methods”. At the current stage and version of your manuscript, I think the test that you have performed is not extensive, please refer to my comments in the “Major comment” section of this review report.

An optional comment:
(this comment is optional and not mandatory; the authors can choose to ignore this comment)

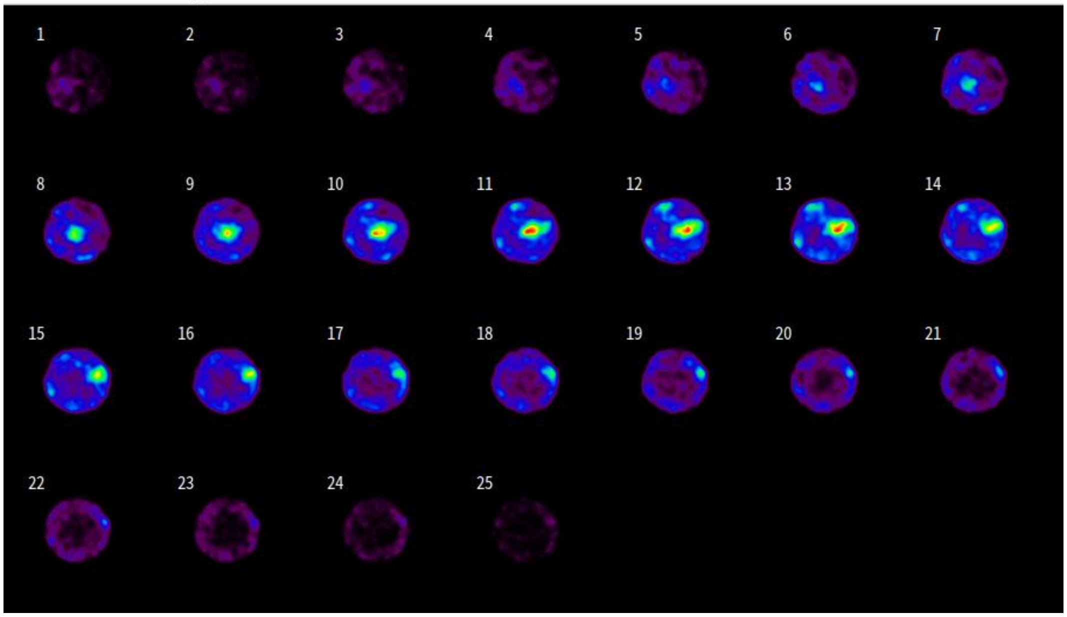

Fig. 1. Reconstructed PET images of mouse head.

If your model can work with .img and .hdr file extensions, I am willing to send you my PET sinogram data that upon reconstruction will reproduce the images shown in Fig. 1. I guess it would be a good test to see how your model behaves when realistic PET measurements are supplied to it.

End of my comments
