# Supplementary material for: Kernel graph filtering—A new method for dynamic sinogram denoising
Source: PLoS One. 2021 Dec 2;16(12):e0260374. doi: 10.1371/journal.pone.0260374 (PMC8638912; doi:10.1371/journal.pone.0260374)
Supplement: Attachment — Submitted filename: Response to Reviewers.pdf [file pone.0260374.s003.pdf]

Responses to Review Comments

(Paper ID: PONE-D-21-19088)

We would like to thank the reviewer for the comments and suggestions which help greatly to improve the quality of this manuscript. We have carefully considered all the comments and suggestions from the reviewer, and have revised the manuscript accordingly. Below is a summary of revision, followed by our point-to-point responses to the reviewer's each comment. The reviewer's comments are quoted in blue color, followed by our responses in black.

1 Revision Summary

To address the reviewer's comments, we have thoroughly revised the manuscript. We have introduced a new Preliminaries section to introduce the basic concepts and technical results on dynamic PET sinograms, graph and graph signal. These were embedded in Method section in original manuscript. In Method Section, we have moved the justifications and explanations on Steps 1-3 of the proposed method to the end of the section and presented them as *Remarks* 1-4. In Result section, we have introduced a new Influence of KGF parameters subsection with new experiment results and explanations in detail. We have rewritten some paragraphs and sentences, and corrected typos in light of the reviewer's comments.

2 Point-to-point responses to the comments of reviewer #1

General comment:
The denoising of PET data has always been the center of attention in the field of medical imaging. There are many studies in the current literature regarding this issue. In this paper, the authors introduced the kernel graph filtering method for denoising PET sinograms. The idea behind this work is interesting. However, the explanation of the theory employed in this method is confusing to me. Some of the parameters are not defined properly and I cannot go through the derivations carefully. The authors need to re-write these once again with clear explanation. I attached my detailed comments. Considering my comments, I recommend major revision of the manuscript before being considered for publication in PLOS ONE.

Thanks for your appreciation of and suggestions on our manuscript. In light of your General comment, we have thoroughly revised the manuscript to improve its presentation, with new structure, new section, new subsection, new paragraphs, new experiment results and analysis, reworded sentences, and clear definitions. We hope that the revised manuscript can be better understood now and meets the standards of PLOS ONE for publication.

Major comments:

1) The methods part is confusing. Not enough physical explanation has been provided for the employed theoretical model.

In light of your comment, we have moved from Method section the introductions on the basic concepts and technical results of dynamic PET sinograms, graph and graph signal, and put them in a new Preliminaries section. In this new section, we have provided more detailed and clearer discussions on the basic physics of PET dynamic sinograms and mathematical definitions of graph, graph signal and graph filter, which are used to develop our new method. In Method Section, we have introduced *Remarks* 1-4 on Steps 1-3 of the proposed method, with an emphasis on the physical explanation and justifications. We believe the revised manuscript has now provided sufficient physical explanation of the proposed method. We hope you would share the same view.

2) Lines 70-71, is index "i" is essentially the scan time? Please explain.

Yes, the index i is related to the scan time but in a bit complicated way. To clarify this we have added in revised manuscript Lines 74-78 the following statement.

"Here, the sinogram frames $\boldsymbol{p}_i$'s are ordered and indexed by the occurrence of their corresponding PET activity time intervals. The activity time interval of $\boldsymbol{p}_i$ precedes that of $\boldsymbol{p}_{i+1}$, and the sinogram data $\boldsymbol{p}_i$ are acquired before the sinogram data $\boldsymbol{p}_{i+1}$ during PET scan process. Hence, a smaller i represents an earlier time interval and a larger i represents a later time interval in the scan. "

3) Line 81, what does the sigma of matrix W represent? Please explain. It is confusing to me.

Sorry about the confusion. It was not the sigma of matrix $\boldsymbol{W}$. It was the sigma (sum) of W_{ij}, and W_{ij} was the ijth element of the matrix $\boldsymbol{W}$ as stated before Line 81. Throughout original manuscript we used boldface letters to represent matrices and vectors, and un-boldface letters with subscripts to represent the elements of matrices and vectors. But we had not explicitly stated these. To avoid confusion, we have refined symbol convention and added the following at the beginning of Preliminaries section in revised manuscript (Lines 64-68) to clarify it.

"Throughout the paper, boldface letters represent matrices and vectors, eg a matrix $\boldsymbol{A}$ and a vector $\boldsymbol{x}$, lower case letters represent scalars (including scalar elements of matrices, vectors and sets), eg an eigenvalue λ and the ijth element a_{ij} of the matrix $\boldsymbol{A}$, and boldface calligraphic letters represent sets, eg the set of $M \times M$-dimensional real valued matrices $\mathcal{R}^{M\times M}$."

Following the refined symbol convention, we have revised the paragraph of original Line 81 to make it clearer. We have replaced W_{ij} (the ijth element of $\boldsymbol{W}$) by w_{ij} and E_{ij} by e_{ij} because they are scalars, and have explicitly defined $d_i = \sum_{j=1}^{n} w_{ij}$ as the degree of vertex v_i according to the graph theory that in a weighted and undirected graph, the degree of vertex v_i is equal to the sum of the weights of all the edges connected to the vertex v_i. The revised paragraph in Lines 104-112 is quoted below.

"Let $G(\mathcal{V}, \mathcal{E}, \boldsymbol{W})$ be a weighted and undirected graph with a set of vertices $\mathcal{V} = \{v_1, v_2, .., v_N\}$, a set of edges $\mathcal{E} \subseteq \mathcal{V} \times \mathcal{V}$ and a weighted adjacency matrix $\boldsymbol{W} \in \mathcal{R}^{N \times N}$. When the vertices v_i and v_j are connected by an edge $e_{ij} \in \mathcal{E}$, the ijth element of $\boldsymbol{W}$, denoted as w_{ij}, is nonzero and equals the weight of the edge, otherwise w_{ij} is zero. Define $d_i := \sum_{j=1}^{N} w_{ij}$ as the degree of vertex v_i, and $\boldsymbol{D} := diag\{d_1, d_2, \cdots, d_N\}$ as the graph degree matrix. The graph Laplacian matrix is given as $\boldsymbol{L} = \boldsymbol{D} - \boldsymbol{W}$, which is symmetric and positive semi-definite. The eigenvalues of $\boldsymbol{L}$ are real valued in an ascending order $0 \leq \lambda_1 \leq \lambda_2 \leq \cdots \leq \lambda_n$ and are defined as the spectra of the graph G [16].

4) Line 86, what is the physical meaning of the parameter "x". Please define and explain.

We have revised the paragraph of original Line 86. In the revised paragraph (Lines 113-120 of revised manuscript), we have clearly defined $\boldsymbol{x}$ as a vector of graph signal, explained its meaning with a simple example, and refined the definition of graph filter as an operator on the graph signal $\boldsymbol{x}$ with the filter output $\boldsymbol{y}$. The revised paragraph is quoted below.

"A signal defined on a graph is called a graph signal. On a graph G with N vertices, a graph signal can be represented by a vector $\boldsymbol{x} \in \mathcal{R}^{1 \times N}$ consisting of the data sampled from the N vertices of G [16]. A simple example is the vector consisting of the intensity values of N pixels in an image modeled by a graph G, with its N vertices representing the N pixels. For a graph signal $\boldsymbol{x} \in \mathcal{R}^{1 \times N}$, an operator $\boldsymbol{F} \in \mathcal{R}^{N \times N}$ that yields $\boldsymbol{y} = \boldsymbol{x}\boldsymbol{F}$ represents a graph filter with the filter output $\boldsymbol{y} \in \mathcal{R}^{1 \times N}$. The adjacency matrix $\boldsymbol{W}$ and the Laplacian matrix $\boldsymbol{L}$ of a graph G are two such operators with the lowpass and highpass natures, respectively, in the graph spectrum domain [21]."

5) Lines 73-75, "The sinogram denoising method to be proposed in this paper does not require specific knowledge about the corrupting noise in the noisy sinograms. Therefore there is no further assumption on $\boldsymbol{p}_i$'s.", meaning is unclear. Please re-write.

We have re-written original Lines 73-75 as shown below to make them clearer.

"The sinogram denoising method to be proposed is based on data adaptive graph filtering and its derivation does not use any specific assumption about the noise in the noisy sinograms $\boldsymbol{p}_i$'s."

6) Equation (1), the Gaussian kernel function, the 2 parameter Gaussian func-

tion could be represented as;

$$G_{2D}(x, y) = \frac{1}{2\pi\sigma^2} e^{\left(-\frac{x^2+y^2}{2\sigma^2}\right)} \quad (1)$$

Comment on the similarities and differences of your Gaussian kernel function and the one We showed above.

Thanks for your comment. There was a typo in original manuscript. $c_{ij} = exp(-||\boldsymbol{p}_i - \boldsymbol{p}_j||_2/\sigma)$ should read $c_{ij} = exp(-||\boldsymbol{p}_i - \boldsymbol{p}_j||^2/2\sigma_1^2)$ and we have corrected it in Eq (1) accordingly. $exp(x)$ here means e^x.

The Gaussian function you showed above is the joint Gaussian probability density function (PDF) of two independent Gaussian variables x and y, with the same expected value 0, the same variance $\sigma_x = \sigma_y = \sigma$, and the scaling factor $\frac{1}{2\pi\sigma^2}$ for the integral of PDF to be 1. Whereas our radial Gaussian kernel function is not a PDF and is used to compute the Gaussian distance between two N-dimensional feature vectors $\boldsymbol{p}_i$ and $\boldsymbol{p}_j$. Hence it doesn't have a scaling factor. Our parameter σ_1 is a free parameter instead of the variance of a random variable and can be varied to tune the width of the kernel. To clarify this we have added in Lines 144-145 of revised manuscript "The σ_1 in (1) is a free parameter that can be tuned if needed to adjust the width of the kernel."

As radial Gaussian kernel function is well explained in previous publications, eg [23,24], we only cite [23,24] in revised manuscript, without comparison with PDF to avoid lengthy discussion. We hope you would agree with us.

7) What does α represent in Eq. (3)? I am again confused.

We understand your question is about the physical meaning of $\boldsymbol{\alpha}$ as well as its definition. To clarify these, we have thoroughly rewritten Step 1 of the proposed method from Eq (3) onwards. We have

1) added a new Eq (3) before the original Eq (3) (now Eq (4)) to explicitly define $\boldsymbol{\alpha}_l$'s as the eigenvectors of the centered kernel matrix $\hat{\boldsymbol{C}}$,

2) stated that $\boldsymbol{\alpha}_l$'s are used together with $\hat{\boldsymbol{C}}$ to extract the principal components of feature vectors $\phi(\boldsymbol{p}_i)$'s,

3) presented the original Eq (3) (now Eq (4)) as a computation algorithm to extract the principal components of feature vectors $\phi(\boldsymbol{p}_i)$'s and hence reduce their dimension, with detail description about the computation procedure, the resulting principal component vectors and their usage,

4) added *Remark* 1 to explain the role of the eigenvectors $\boldsymbol{\alpha}_l$'s in kernel PCA with an equivalent expression of Eq (4) (original Eq (3)) from kernel PCA theory [22,24].

The details of above can be found in Lines 149-163 and Lines 227-234 of revised manuscript.

8) Equation (5), what γ actually does here?

As quoted below, we have simplified original Eq (5) (Eq (6) in revised manuscript) and its description, and replaced γ by the total number of sinogram frames N in the equation. We have provided a mathematical derivation

of new Eq (6) and explained its physical meaning in newly added *Remark* 2 in Lines 235-254 of revised manuscript.

"The number of the nearest neighbors in $k\text{nn}_i$ is determined by

$$k_i = r(N||\boldsymbol{p}_i||_1/||\boldsymbol{p}_N||_1),\ i = 1, 2, \cdots, N, \tag{6}$$

where $\|\boldsymbol{p}_i\|_1$ is the 1-norm of the sinogram vector $\boldsymbol{p}_i$, and $r(\cdot)$ is the operator of rounding to the nearest integer."

9) Lines 141-144, "At smaller i's, the energy is low and energy variations are large, we use smaller $k\text{nn}_i$ to include fewer neighbour sinograms, namely, fewer nonzero a_{ij}'s. At larger i's, the energy is high and energy variations are small, we use larger $k\text{nn}_i$ to include more neighbour sinograms, namely, more nonzero a_{ij}". I am again confused here, and I cannot follow the derivations. Please re-write.

Thanks for your comment. To better explain the above statement, we have thoroughly rewritten the Noisy dynamic sinograms subsection (Lines 79-92 of revised manuscript) and added a new *Remark* 2 (Lines 235-254 of revised manuscript). We have used the 1-norm of sinograms in both these places to quantify their energy and hence made the explanation quantitative to help understanding.

10) Please discuss in detail and in a clear manner, the difference between your work and previous work cited in Ref.[10] by Wang et. al.

The essentially difference between our work and previous work (Ref. [10], KEM) is that ours is a pre-denoising algorithm, while KEM is a reconstruction algorithm. Our method is intended for use with existing reconstruction algorithms including KEM in [10]. The reason we use our method in KEM is to show the improvement of our pre-denoising method on the state-of-art reconstruction method KEM. To highlight the different functions of our method and KEM of [10] we have revised

the first paragraph of Step 3 (Lines 197-203 of revised manuscript): "The kernel graph filtered sinograms $\hat{\boldsymbol{P}}^*$ can be used as input data in any existing dynamic PET image reconstruction methods to improve their image quality. In this step, we only use $\hat{\boldsymbol{P}}^*$ in two popular iterative reconstruction methods: the traditional method of Maximum-Likelihood Expectation Maximization (MLEM) [26,27] and the recent method of Kernalized Expectation Maximization (KEM) [10]. The intention is to show the image quality improvement from using $\hat{\boldsymbol{P}}^*$, instead of proposing a new method for dynamic PET image reconstruction."

the last sentence of Discussion (Lines 535-537 of revised manuscript): "It is therefore fair to say that the KGF denoised sinograms can significantly improve the image quality of KEM and MLEM reconstructions, especially at the low count frames."

the first sentence of Conclusion (Lines 539-541 of revised manuscript): “A novel kernel graph filtering method has been proposed to effectively reduce the noise in dynamic PET sinograms. It can be used with the existing methods for sinogram based dynamic PET image reconstruction to improve the quality of reconstructed image.”

11) The PET data you have used are actually simulated and you have introduced Poisson noise, the method seems to work well. However, it is highly recommended to use realistic experimental data. Please use some realistic measurement examples and compare.

Thanks for your suggestion. The in-vivo data used in our paper is indeed realistic experimental data. As described in Tests on in-vivo data subsection, the in-vivo patient data is from [33] (authored by our partner in Monash Biomedical Imaging, Monash Univ, Australia). The data was acquired from volunteers with 90 min on a Siemens (Erlangen) Biograph 3 Tesla molecular MR (mMR) scanner. Compared with other denoising methods, the proposed method has better visual performance in the real life application. To highlight this we have changed the title of in-vivo test subsection to “Tests on real life in-vivo data” (Line 465 of revised manuscript).

12) Lines 267-275, the authors used some parameter values. I am again confused how these values are chosen? Some sort of justification would be needed for the use of these values. If in case, it is really not possible to justify and these values are chosen in a random fashion, the you need to perform sensitivity study by changing the values of each of these parameters and investigate their respective influence on the final results.

In light of your advice, we have added in a new subsection: Influence of KGF parameters to analyze the sensitivity of three parameters (the number of kernel principle component d in Step 1 Eq. (4), σ_2 of Gaussian kernel function in Step 2 Eq. (5), constant ϵ in Step 2 Eq. (9)), respectively in the proposed method. We have stated these in Result section (Lines 440-463 revised manuscript).

I have some PET data of mouse head, there are namely two file extension .img and .hdr, does your program model takes .img and .hdr? please confirm this.

Thanks for your offer. Unfortunately our model can’t use .img and .hdr file extensions. As reported in Tests on real life in-vivo data subsection, we have already tested our method on real patient PET scan data.

Minor comments:

1) Lines 14-15, “For example, when other imaging modalities, such as magnetic resonance (MR) or computed tomography (CT), are incorporated, some bone or MR-only lesion [4] originally nonexistent in the PET image may be introduced.”. meaning unclear, please re-write.

We have re-written this sentence.

2) Line 24, “correlated and the is very difficult to reduce.” Remove “the”.

We have made the corrections.

3) Line 31, "to suppress, and spur the development", please consider replacing the word "spur" with something else.

We have replaced 'spur' with 'stimulate'.

4) Lines 31-32, "It has been shown that pre-denoising in sinograms more efficient post-denoising in PET images". Wrong grammar, re-write.

We have re-written this sentence.

5) Regarding MLEM method, please consider citing more recent papers. Example; Studies on unfolding energy spectra of neutrons using maximum-likelihood expectation-maximization method. Nuclear Science and Techniques. 2019 Sep;30(9):1-0.

Thanks for your advice. We have added this reference in revised manuscript. (Line 200)

6) Lines 227-228, "To get dynamic PET images, the time activity curve (TAC) in Fig 1 (b) was filled into corresponding tissues to produce the noiseless ground truth images". Please explain how you have obtained the time-activity curves in your work.

In our work, the time activity curve (TAC) was generated using three-compartment model [30]. The kinetic parameters were from [31]: $K_1 = 0.116\ ml/g/min$, $k_2 = 0.254\ min^{-1}$, $k_3 = 0.116\ min^{-1}$ and $k_4 = 0.011\ min^{-1}$ for gray matter, $K_1 = 0.059\ ml/g/min$, $k_2 = 0.149\ min^{-1}$, $k_3 = 0.090\ min^{-1}$ and $k_4 = 0.013\ min^{-1}$ for white matter, $K_1 = 0.089\ ml/g/min$, $k_2 = 0.269\ min^{-1}$, $k_3 = 0.135\ min^{-1}$ and $k_4 = 0.015\ min^{-1}$ for the lesion. The scanning sequence contained a total of 24 frames. We have added these descriptions to Lines 286-291 in revised manuscript.

7) Lines 269-270, "For fair comparison, these parameters were tuned differently on the sinograms data". How did you tune these parameters? Please elaborate.

We have rewritten this sentence as below to elaborate our tuning method. "For fair comparison, we tune the parameters of each method on the sinogram data to attain its lowest sinogram MSE value, and use the corresponding parameters for the method. Using this tuning method we find the best parameters for each evaluated method."

We have detailed the best parameters for each evaluated method obtained from the tuning in Lines 333-336 of revised manuscript.

8) Fig. 6, Please consider changing line and symbol colors for different method, it is hard to differentiate these. Check for similar issue in other plots.

Thanks for your advice. We have changed the line styles and colors in Fig 6 and Fig 9.

9) In conclusion, Lines 447-448, Extensive simulation studies and tests on in-vivo dynamic PET data have shown the efficacy and advantages of the proposed method over the existing methods. At the current stage and version of your manuscript, I think the test that you have performed is not extensive, please refer to my comments in the Major comment section of this review report.

Thanks for your suggestion. According to your advice, we have added the parameter analysis in revised manuscript (Lines 440-463).

An optional comment: (this comment is optional and not mandatory; the authors can choose to ignore this comment)

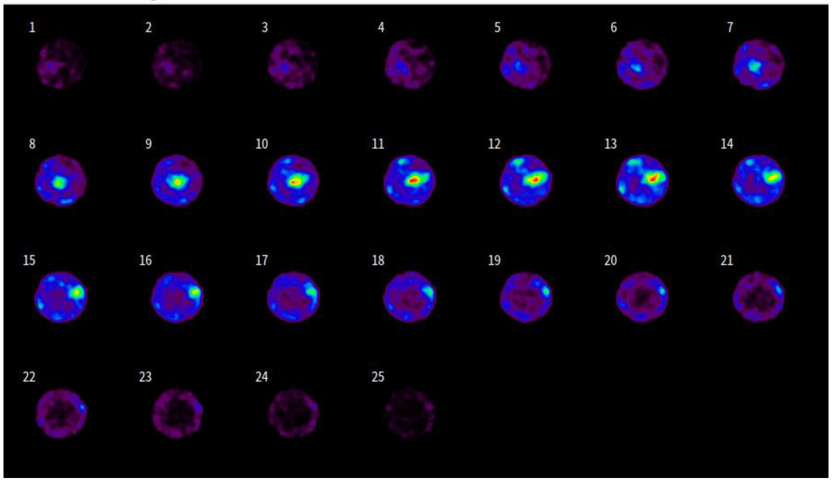

Figure 1: Reconstructed PET images of mouse head

If your model can work with .img and .hdr file extensions, I am willing to send you my PET sinogram data that upon reconstruction will reproduce the images shown in Fig 1. I guess it would be a good test to see how your model behaves when realistic PET measurements are supplied to it.

Thanks for your offer. Unfortunately our model can't use .img and .hdr file extensions. As explained before and also reported in Tests on real life in-vivo data subsection, we have already tested our method on real patient PET scan data.
